# Perceptions and factors associated with the uptake of the community client-led antiretroviral therapy delivery model (CCLAD) at a large urban clinic in Uganda: a mixed methods study

**DOI:** 10.1186/s12913-023-10182-7

**Published:** 2023-10-26

**Authors:** Happy Annet Gasaatura Walusaga, Lynn M Atuyambe, Martin Muddu, Ruth Mpirirwe, Joan Nangendo, Dennis Kalibbala, Fred C. Semitala, Anne R. Katahoire

**Affiliations:** 1 Makerere University Joint AIDS Program (MJAP), Kampala, Uganda; 2 Department of Community Health and Behavioural Sciences, College of Health Sciences, Makerere University School of Public Health, Kampala, Uganda; 3 Department of Medicine, College of Health Sciences, Makerere University, Kampala, Uganda; 4 School of Statistics and Applied Economics, Kampala, Uganda; 5 Child Health Development Centre, Makerere University College of Health Sciences, Kampala, Uganda

**Keywords:** Antiretroviral therapy, Streamlined model, Patient perceptions, HIV care, Differentiated service delivery models.

## Abstract

**Introduction:**

Community Client-Led ART Delivery (CCLAD) is a community HIV care model. In this model, a group of persons living with HIV (PLHIV) in a specific location, take turns going to the HIV clinic to pick up Antiretroviral Treatment refills for members. The uptake of this model, however, remains low despite its improvements in patient retention. In this study, we explored PLHIV’s perceptions of this model and identified the factors associated with its low uptake.

**Methods:**

This was a mixed methods study based on a retrospective review of records of PLHIV and in-depth interviews. We reviewed the medical records of people receiving ART to determine their current model of ART delivery and conducted in-depth interviews with 30 participants who were eligible to be enrolled in the CCLAD model at the Mulago ISS clinic. We performed logistic regression to identify factors associated with the uptake of the CCLAD model and inductive thematic analysis to explore PLHIV’s perceptions of the CCLAD model.

**Results:**

A total of 776 PLHIV were sampled for the study, 545 (70.2%) of whom were female. The mean age (standard deviation) was 42 (± 9.3) years. Overall, 55 (7.1%) received ART using the CCLAD model. Compared to other ART-delivery models, CCLAD was associated with being on ART for at least eight years (AOR 3.72; 95% CI: 1.35–10.25) and having no prior missed clinic appointments (AOR 10.68; 95% CI: 3.31–34.55). Mixed perceptions were expressed about the CCLAD model. Participants interviewed appreciated CCLAD for its convenience and the opportunities it offered members to talk and support each other. Others however, expressed concerns about the process of group formation, and feeling detached from the health facility with consequences of lack of confidentiality.

**Conclusion:**

The current uptake of the CCLAD model is lower than the national recommended percentage of 15%. Its uptake was associated with those who had been in care for a longer period and who did not miss appointments. Despite CCLAD being perceived as convenient and as promoting support among members, several challenges were expressed. These included complexities of group formation, fear of stigma and feelings of detachment from health facilities among others. So, while CCLAD presents a promising alternative ART delivery model, more attention needs to be paid to the processes of group formation and improved patient monitoring to address the feelings of detachment from the facility and facility staff.

**Supplementary Information:**

The online version contains supplementary material available at 10.1186/s12913-023-10182-7.

## Introduction

HIV/AIDS remains a major global public health concern affecting approximately 38 million people worldwide, 55% of whom are from East and Southern Africa [1]. In 2014, the Joint United Nations Programme on HIV/AIDS (UNAIDS) launched the updated 95-95-95 targets as a strategy to end the epidemic by 2030 [2]. The strategy aims to ensure that 95% of people living with HIV (PLHIV) know their status, 95% of those diagnosed receive ART, and 95% of those receiving ART achieve viral suppression. Although Uganda has made commendable progress towards these targets [2], only 78% of people receiving ART are virally suppressed. Retention in HIV care is a crucial step in achieving viral load suppression. PLHIV in Uganda face various access barriers to routine regular clinic attendance. These include; HIV stigma and long distances to health facilities [3]. In 2017, the Uganda Ministry of Health (MoH) launched differentiated service delivery (DSD) models for HIV care to minimize patient-level barriers to accessing HIV care and reduce congestion in health facilities.

By 2019, four DSD models for clinically stable clients were being implemented in Uganda **(**Table 1). The implementation of the CCLAD model was intended to (i) bring services closer to people, (ii) reduce HIV stigma, (iii) streamline clinic visits, (iv) address limited facility infrastructure, and (v) reduce health worker overload [3]. The ultimate goal of DSD models is to improve retention and viral suppression of PLHIV in care. Of the different DSD models, CCLAD was the least utilized, at 6% [4, 5], which was less than the 15% recommended by MoH [6]. A review of clinic data at ISS Clinic in Mulago revealed that approximately 7.1% of PLHIV were enrolled in the CCLAD model [6]. This study aimed to determine the factors associated with the uptake of CCLAD and explore PLHIV’s perceptions of this model of care at the Mulago ISS Clinic.

**Table 1:** Description of the differentiated ART delivery models in Uganda [6]

Model	Description
1. Fast-Track Drug Refills (FTDR)	ART-experienced people with HIV pick their antiretrovirals at the facility pharmacy for three or more months.
2. Facility -Based Groups (FBG)	ART-experienced people with HIV are served in a group at the facility. These are support groups for stable or unstable/complex.
3. Community Drug Distribution Points (CDDP)	People pick their drugs and receive a follow-up clinical evaluation by healthcare providers at their preferred outreach point.
4. Community Client-Led ART model (CCLAD)	Three to six people from the same community voluntarily form groups and take turns collecting drug refills from the facility.

## Methods

### Study Design and setting

We employed a mixed-methods convergent parallel design. We collected quantitative and qualitative data concurrently but analyzed them separately during the same timeframe. The qualitative sample was a sub-sample of the quantitative. This design enabled us to collect complementary data to gain a more in-depth understanding of the perceptions and factors associated with the uptake of the CCLAD model. The results were integrated in the discussion. We analyzed secondary data from patients’ clinical charts at the Mulago HIV clinic to identify factors associated with using the CCLAD. We also conducted in-depth interviews with a purposively selected group of participants who were eligible for the CCLAD to explore their perceptions of the model.

The Mulago ISS clinic is the largest urban HIV facility in Uganda, providing comprehensive HIV services to approximately 16,500 PLHIV. It is located within Mulago National Referral and Teaching Hospital Complex in Kampala, Uganda’s capital city. The clinic has implemented both facility-based and community-based DSD models since 2017. It was selected for this study because of the high volume of clients served by the clinic from different parts of the country.

### The CCLAD model at the Mulago ISS clinic

In CCLAD, a group of persons living with HIV (PLHIV) in a specific location, take turns going to the HIV clinic to pick up Antiretroviral Treatment refills for members. For lay group leaders to manage groups, the Ministry of Health recommends that membership of CCLAD range between 3 and 6 members. After every three months, one group member picks antiretrovirals for the rest of the members. All group members are expected to visit the health facility twice annually for comprehensive evaluation and viral load testing. Through its community department, the Mulago ISS clinic has a tracking system that tracks which group member has represented the others and who will come next. After the group member has delivered ART refills to the group members, they report to the clinic through phone calls to update the electronic medical records and patient charts.

### Study population and participants

The study population was comprised of clinically stable PLHIV 18 years of age and above who had received ART at the Mulago ISS clinic for two years or more as of September 2020.

### Inclusion and exclusion criteria

The quantitative component included all active adults (≥ 18 years**)** in care with a documented suppressed viral load in the previous 12 months. However, we excluded participants with a documented transfer request within one month following the data extraction date and those with a known psychiatric illness or tuberculosis treatment.

Participants were eligible for the qualitative component of the study if they were; sampled for enrolment in the quantitative study, enrolled in one of the three DSD models for stable clients (the CCLAD, FBG, or FTDR), and able to speak and understand English or Luganda (the most widely spoken language in this part of the country).

### Sample size estimation

The sample size for the quantitative component of the study was calculated using the Fleiss formula, considering a 50% uptake of CCLAD among females and 60% for males. The final estimated sample size was 816 participants.

$${N_{Fliess}}{\rm{ = }}\frac{{[{z_{\alpha /2}}]\sqrt {(r + 1)p(1 - p)} + {z_\beta }\sqrt {r{p_0}(1 - {p_0}) + {p_1}(1 - {p_1})} {]^2}}}{{r{{({p_0} - {p_1})}^2}}}$$

Where; Zα/2 is the standard normal value corresponding to the 5% level of significance = 1.96, Z_β_ is the standard normal value corresponding to the power of 80%, the critical value = 0.84, r- is the ratio of exposed to unexposed (r) = 1, P_0_ is the percent of women choosing community-based model = 50%, P_1_ is the percent of men choosing community-based model = 60%, and P is the average value of P_0_ and P_1_ = 55%.

**Fig. 1:**
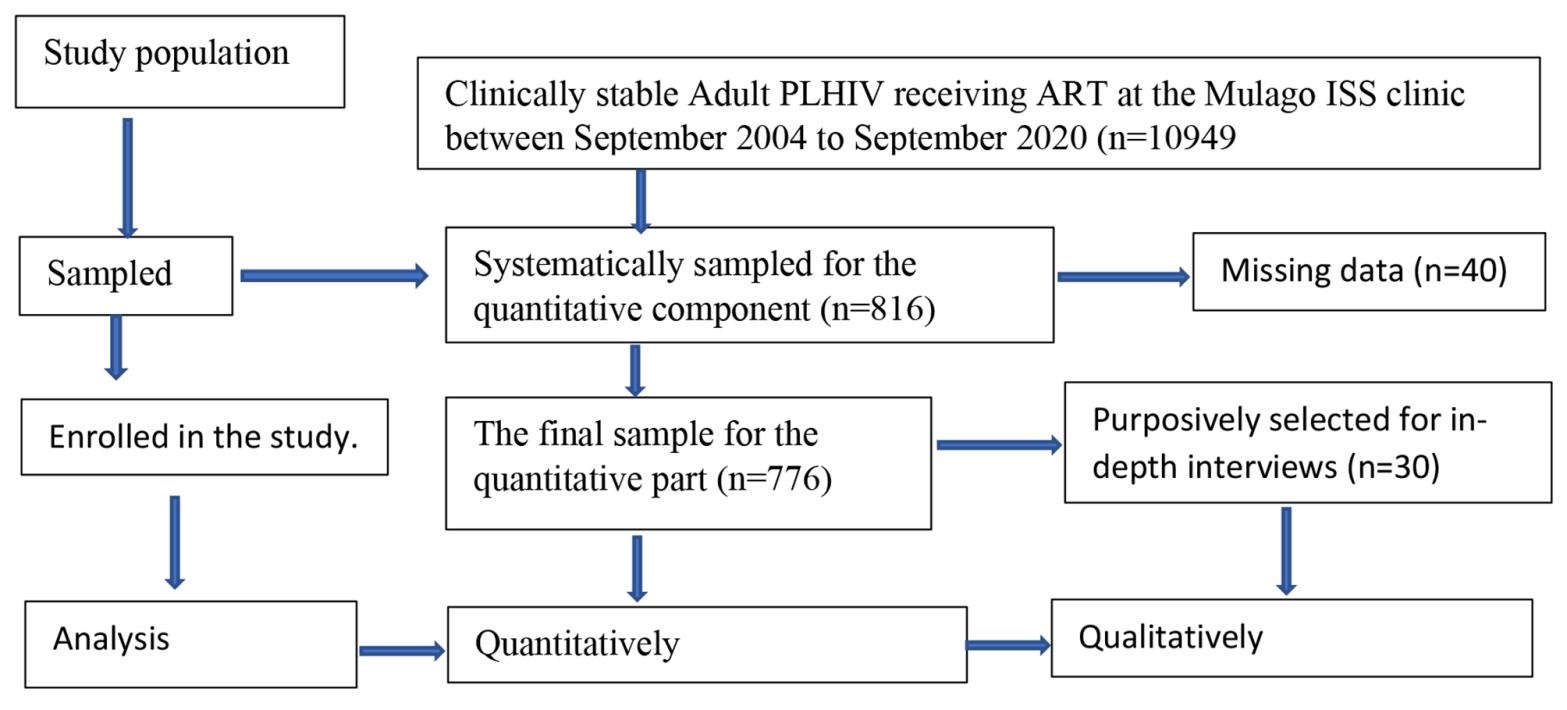
Study flow diagram illustrating participant recruitment into the study; adopted from [13] and modified for this study

Using an Internet-based Research Randomizer tool [7], we randomly selected 816 participants from the 10,949 clinically stable patients registered in the clinic. Participants were sampled electronically from the clinic’s Open Electronic Medical Records System (EMRS). Although the estimated sample size was 816 people, 40 of the selected records had missing data and were dropped, leaving 776 as our final sample size (Fig. 1).

For the qualitative component of the study, we purposively sampled a sub-sample of those whose records were included in the quantitative component. These included adult PLHIV who had spent two or more years receiving care through one of the DSD models. For maximum variation, participant’s social-demographic characteristics, stability on ART, viral suppression, DSD model, and disclosure status were taken into consideration. Study participants were invited to participate and recruited until no new information was obtained. In total, we conducted 30 in-depth interviews.

### Data collection

In the quantitative component of the study, we extracted clinical and demographic data of the enrolled active HIV patients from the clinic EMR. These data included age, education level, sex, marital status, monthly income, and location. The other extracted data were on the client duration on ART, HIV status disclosure, viral load, presence of HIV comorbidities, and World Health Organisation (WHO) clinical staging.

For the qualitative component, we developed and piloted an in-depth interview schedule which guided data collection process. Some examples of questions asked included: *What are the advantages and disadvantages of receiving care through CCLAD?*, *We have observed low adoption of the CCLAD delivery model in this clinic. What factors could be contributing to this?* (Supplementary file 1). We collected data on perceptions of the CCLAD model of care through in-depth interviews. Participants were interviewed in English or Luganda, and the sessions lasted 35 to 45 min. Some participants (n = 5) preferred to be interviewed at a private place of their choice within their communities, while the rest (n = 25) were interviewed at the Mulago ISS clinic.

### Data Management and Analysis

We downloaded data from EMRs into a Microsoft Excel spreadsheet for cleaning and assigned each participant a unique study identification number. Data were then exported into STATA 16 (Stata Corp, College Station, TX, USA) for analysis. Descriptive statistics were expressed as means (± standard deviation) for numerical variables, while categorical variables were expressed as percentages. The uptake of the CCLAD model was determined as the proportion of participants who receive their ART refills through the CCLAD model.

We used logistic regression to determine the unadjusted and adjusted odds ratios (95% confidence intervals, CI) of the covariates associated with being enrolled in the CCLAD model. The overall prevalence of CCLAD utilization was less than 10%, making the odds ratio the best measure of association [7]. All covariates with a P ≤ 0.25 at the bivariate analysis level were considered at the multivariate analysis level. Covariates with P < 0.05 in the final adjusted model were considered to be associated with being enrolled in the CCLAD model.

For the qualitative data, the audio-recorded interviews were transcribed verbatim, and translated into English. The data were coded using ATLAS. ti version 22.2.5. and analyzed using inductive thematic analysis [8]. In the first stage, two authors experienced in qualitative research conducted a thorough reading of three transcripts separately to identify potential codes. They then jointly worked together to generate a codebook with code definitions. The codes were then categorized to form major themes that summarised participants’ perceptions of the CCLAD model.

### Integration of the qualitative and quantitative results

The two data sets were complimentary in nature. The quantitative results highlighted the factors associated with the uptake of the CCLAD model, while the qualitative findings provided client perceptions of the model. These results were integrated at the discussion level.

### Ethics and Consenting

The study was approved by The AIDS Support Organization (TASO) Research and Ethics Committee (TASO REC 031/2021-REC-009) and the Uganda National Council for Science and Technology (UNSCT-SS957ES). We also obtained administrative clearance from the leadership of the Mulago ISS clinic to access and abstract data from the clinic’s medical records. This was a purely observational study with no experiments and no removal of human tissue samples. All study methods followed the relevant guidelines and regulations of TASO REC–009 and UNSCT-SS957ES.

We obtained a consent waiver to use the clients’ medical data (secondary data) for quantitative data collection. The participants who participated in the in-depth interviews provided written informed consent to participate in the interviews and for them to be recorded. For participants who could not read or write, informed consent was obtained from their legal guardians. The study was fully explained to potential study participants, either in Luganda for the non-English speaking or in English for the English-speaking. This allowed participants to make an informed decision for participation in the study. Participants were free to withdraw from the study at any time if they wanted, without any consequence on their care at the clinic. Confidentiality was ensured by replacing names with unique study identification numbers. Participant data (audio recordings and the abstracted database) were stored on password-protected computers.

## Results

### Demographic characteristics of the study participants

Of the 816 participants selected for participation, 40 patients were excluded due to missing data. A total of 776 respondents participated in the study, 572 (of whom were female (n=, 70.1%). More than a third, 319 (39.1%) had attained primary education, and nearly half, 401 (49.1%) were married. The mean age (± standard deviation) age was 42 (± 9.3) years. About a fifth 182 (22.3%) of the participants had a monthly income of between 50,000 and 100,000 UGX (13–27 USD). Three quarters 650 (75.4%) of them had been on ART for more than five years (Table 2).

**Table 2:** Demographic Characteristics of Study Participants

Participant information	Percentage (n)
Age, Mean (SD)	42(± 9.3)
18–24 years	1.6 (13)
25–31 years	10.5 (86)
32–38 years	24.3 (198)
39–45 years	29.7 (242)
46–52 years	21.1 (172)
>52 years	12.8 (105)
Sex	
Female	70.1 (572)
Male	29.9 (244)
Education level*	
None	3.9 (32)
Primary Level	39.1 (319)
Secondary Level	33.5 (273)
Tertiary Level	6.4 (52)
Marital status**	
Divorced/Separated	27.6 (225)
Married	49.1 (401)
Single	12.9 (105)
Widowed	8.4 (69)
District of residence	
Kampala	60.8 (477)
Wakiso	34.6 (272)
Others	4.6 (36)
Monthly income (UGX)	
None	17.9 (146)
Less than 50,000	9.9 (81)
50,000–100,000	22.3 (182)
Above 100,000	31.5 (257)
Co-morbidities	
DM only	0.1 (1)
DM with hypertension	0.1 (1)
Hypertension	6.2 (50)
The most recent HIV viral load result	
<1000 copies/mL	98.5 (804)
≥1000 copies/mL	1.5 (12)
WHO clinical staging at ART initiation	
Stage 1	44.1 (360)
Stage 2	29.5 (241)
Stage 3	21.2 (173)
Stage 4	5.2 (42)
Duration on ART	
<5 years	24.6 (201)
5–7 years	30.6 (250)
8–10 years	24.6 (201)
11–13 years	20.2 (164)

There were no significant differences between the excluded participants(n = 40) and those included in the analysis (Table 3).

**Table 3:** Characteristics of 40 participants who were excluded from the quantitative analysis

	Included	Excluded	P-value
N (%)	776 (95.1%)	40 (4.9%)	
Age	42.282 (9.282)	39.800 (8.727)	0.099
Sex			
Female	545 (70.2%)	27 (67.5%)	0.713
Male	231 (29.8%)	13 (32.5%)	
Education level			
None/primary	465 (59.9%)	26 (65.0%)	0.522
Secondary/tertiary	311 (40.1%)	14 (35.0%)	
Marital status			
Divorced/separated	216 (28.4%)	9 (23.1%)	0.617
Married/cohabiting	380 (49.9%)	21 (53.8%)	
Single	98 (12.9%)	7 (17.9%)	
Widowed	67 (8.8%)	2 (5.1%)	
District			
Other	34 (4.5%)	2 (5.6%)	0.776
Kampala	715 (95.5%)	34 (94.4%)	
Income (UGX)			
None	137 (21.7%)	9 (25.7%)	0.398
50,000–100,000	253 (40.1%)	10 (28.6%)	
Above 100,000	241 (38.2%)	16 (45.7%)	
WHO HIV clinical stage			
Stage 1	341 (43.9%)	19 (47.5%)	0.943
Stage 2	229 (29.5%)	12 (30.0%)	
Stage 3	166 (21.4%)	7 (17.5%)	
Stage 4	40 (5.2%)	2 (5.0%)	
Disclosed to someone			
No	128 (16.5%)	8 (20.0%)	0.562
Yes	648 (83.5%)	32 (80.0%)	
Ever missed appointment			
No	498 (64.2%)	21 (52.5%)	0.134
Yes	278 (35.8%)	19 (47.5%)	
Disclosure			
Partner	301 (46.5%)	14 (43.8%)	0.756
Relative	182 (28.1%)	9 (28.1%)	0.996
Friend	135 (20.8%)	4 (12.5%)	0.254
Children	31 (4.8%)	0 (0.0%)	0.205
Parent	53 (8.2%)	4 (12.5%)	0.389

Key: where N = total number of participants

### Uptake of the CCLAD model

Less than a tenth of the 776 participants, 55 (7.1%, 95% CI:5.5 − 9.1%) used the CCLAD model for ART refills. The FTDR model had the highest uptake (n = 708, 86.8%, 95% CI:84.3-88.9%), followed by the FBG model (n = 13, 1.6%; 95% CI:0.9-2.7%) (Table 4). The factors associated with the current uptake of CCLAD were having been on ART for more than eight years (AOR 3.72; 95% CI: 1.35–10.25) and having not missed any clinic appointments (AOR 10.68; 95% CI: 3.31–34.55).

**Table 4:** Utilisation of the CCLAD model (n = 776)

Model	Frequency	Percentage	(95% confidence interval)
CCLAD	55	7.1	(5.5-9.1%)
Other models	721	92.9	(90.8–94.5)

### Factors associated with the use of the CCLAD model

In the final multivariate analysis, having been on ART for 8 to 10 years and having no previous missed clinic appointments were associated with the uptake of the CCLAD model. Compared to participants on ART for less than five years, clients who were on ART for at least eight years were 3.57 times more likely to use the CCLAD model (aOR 3.57; 95% CI 1.10-11.56). In addition, participants who had no prior missed appointments were 13.27 times more likely to use the CCLAD model of care (aOR 13.27, 95% CI: 3.17–55.45) (Table 5).

**Table 5:** Factors associated with the use of the CCLAD model of care

Covariates	Other DSD model	CCLAD model	uOR (95% CI)	aOR (95% CI)
Age			1.01(0.97–1.03)	
18–24 years	13(1.8)	0(0)		
25–31 years	75(10.4)	3(5.4)	1	
32–38 years	168(23.3)	17(30.9)	2.53(0.71–8.89)	
39–45 years	217(30.1)	16(29.1)	1.84(0.52–6.50)	
46–52 years	154(21.4)	11(20.0)	1.78(0.48–6.59)	
>52 years	94(13.0)	8(14.6)	2.12(0.54–8.30)	
Sex				
Male	216(93.5)	15(6.5)	1	1
Female	505(92.7)	40(7.3)	1.14(0.62–2.10)	1.18 (0.57–2.39)
Education level				
Primary or less	426(91.6)	39(8.4)	1.69(0.93–3.08)	1.71(0.86–3.39)
Secondary or more	295(94.9)	16(5.1)	1	1
Marital status				
Divorced/separated	200(92.6)	16(7.4)	0.96(0.51–1.83)	0.90(0.43–1.84)
Married	351(92.4)	29(7.6)	1	1
Single	94(95.9)	4(4.1)	0.52(0.17–1.50)	0.22(0.03–1.77)
Widowed	61(91.0)	6(9.0)	1.19(0.47–2.98)	1.39(0.47–4.14)
Duration on ART				
<5 years	179(97.3)	5(2.7)	1	
5–7 years	224(92.6)	18(7.4)	2.88(1.04–7.90)*	2.31(0.75–7.10)
8–10 years	173(90.6)	18(9.4)	3.72(1.35–10.25)*	3.57(1.10-11.56)*
11–13 years	145(91.2)	14(8.8)	3.45(1.22–9.82)*	3.21(0.94–10.97)
Disclosed to someone				
No	121(94.5)	7(5.5)	1	1
Yes	600(92.3)	48(74)	2.39(0.61–3.12)	0.56(0.20–1.52)
Ever missed a clinic appointment				
No	446(89.6)	52(10.4)	10.68(3.31–34.55)^***^	**13.27(3.17–55.45)** ^***^
Yes	275(98.9)	3(1.1)	1	1

Key: * p < 0.05, ** p < 0.01, *** p < 0.001; CCLAD- client-led antiretroviral therapy (ART) delivery (CCLAD), uOR -unadjusted odds ratio; aOR-adjusted odds ratio

### PLHIV perceptions of the CCLAD model of care

Seven main themes emerged from the analysis of the qualitative data, concerning the CCLAD model. PLHIV’s perceptions of the CCLAD model were varied. Some appreciated the model because it promotes social support and is convenient in terms of saving transport costs. Others however highlighted some challenges with the model. These included; complexities relating to group formation, lack of trust, stigma due to unintended disclosure, fear of detachment from healthcare providers and facilities and the consequences of this including lack of privacy, and limited opportunity for negotiation among others.

#### Social support

Participants perceived the CCLAD model not just as an avenue for receiving ARVs in the community but also as an opportunity to talk and support each other. Through the formation of groups, PLHIV in one location were able to come together and support each other during difficult times.

*“It helped us so much during the lockdown because one would at least use a bicycle and distribute cassava (local food) to fellow group members. One would even call and say that they are not doing well financially, and the other would send maybe Ush.5000. The group has been so helpful when transport is on lockdown. A fellow member with a motorcycle would advise us to take our cards to a central point, from where he would pick them and get us medicine”*– a 56-year-old male.

*“We talk and we share experiences about how we take our medicine, the time we take it and how it affects us” 44-year-old female.*

#### Convenient in terms of transport costs

The CCLAD model was also perceived to be convenient in that PLHIV were able to save on transportation costs, a key barrier to care engagement. The process of picking ARVs in turns enables one to pay transport costs once in six months or annually as opposed to clients individually picking up their refills every three months.

*“I would recommend it (CCLAD); it is okay. It has no problem. You may find that someone saves transport even five times when one of them picks, which is beneficial. Transport issues are sorted”* – a 54-year-old male.

#### Complexities relating to group formation

While some perceived CCLAD as an opportunity for clients to talk and support each other, others expressed concerns about the process of group formation.

*“To join the community model, you need to know each other and how many you are, all of those who take the HIV medicine, yet I also don’t know.”* – 51-year-old male.

PLHIV are allowed to identify each other or can be supported by a healthcare provider to identify those from the same location and bring them together. While this approach strengthens cohesiveness, the participants felt that they did not know each other well enough to be able to connect. Others did not want to know each other despite receiving care from the same facility for fear of unintentionally disclosing their HIV status.

*“That’s why you see most people prefer coming here mostly me because I take the clinic to be like a bank because when you go to the bank to borrow money, no one will know apart from me and the bank. So, I come here personally and pick my drugs, no one gets to know that am sick apart from my people and or unless you inquire from me and then I tell you”-59-year-old-male.*

In addition, another respondent expressed fear about unintended disclosure. The CCLAD model requires members to introduce themselves to one another, which necessitates disclosure that is perceived as undesirable by some since they all live in the same locality. Several participants felt that receiving individual care from a health facility promotes confidentiality rather than being exposed in the community where one stays.

*“…most clients find the CCLAD model challenging because they do not want other clients to know about their HIV status. You may have a friend, but each one does not know the other’s HIV status. The other person may not know if I am on medication or not. If you meet at the drug pickup point, everyone is suspicious of the other…!”*– 32-year-old female.

#### Lack of Trust

In addition to not knowing each other, respondents expressed mistrust of colleagues’ ability to deliver the drugs safely as professional health workers would. They feared their colleagues would pick the wrong ART regimens or even sell them. They particularly questioned the capacity of their colleagues to store the medicines well until the members pick them up, especially if given multi-month refills:

*“About these people going to the community (the CCLAD model), I don’t like it so much because first, trusting that person! But then, is that person also qualified? Does he have the system of picking and storing that medication safely?” –* 58-year-old male.

#### Stigma due to unintended disclosure

Having to identify each other or being facilitated by health care providers to form groups risks disclosure of one’s HIV status to group members. Participants expressed their fear of being identified as HIV-positive if they belonged to a CCLAD.

*“Where I stay, it’s very hard; (there’s) a lot of rumourmongering. I do not want to attach myself to any group to deliver my medicine… My children are still young. I do not want them to know about my condition” a 39-year-old female.*

Participants expressed fear that members may disclose their status to the community. Indeed, some participants reported the fear of disclosure as the reason for not participating in CCLAD:

*“Most people chose to stay in the clinic model because they feared that in the case of a misunderstanding, others would talk about them in the community. For that reason, many did not join”* – a 51-year-old female.

#### Fear of detachment from healthcare providers and health facilities

PLHIV were not only concerned about unintended disclosure but also felt that the model could potentially detach them from their HIV clinic and health providers. They feared that this would also; deny them access to other services available at the clinic including, consultation with trained healthcare providers and negotiating for longer ART refills. PLHIV valued the relationships they had built with their healthcare providers over a long time. They felt that their physical interaction with providers was curtailed by the CCLAD model:

*“I prefer to come to the clinic myself and pick up my drugs. And the other thing is I want to come and talk to my doctor and be able to express my health concerns because if I am to send someone, he won’t know my health concerns.” –* 25-year-old female.

Participants also shared concerns about the likelihood of not receiving quality services as those who go to the facility.

*“For me, I want to come to the clinic; Because “eyetukidde tanywa matabangufu” translated as “One who personally goes to a water well does not drink muddy water”* 25-year-old female.

This literally meant that the one who goes to the facility is unlikely to get poor-quality services.

Another participant when asked why she did not join CCLAD, yet she was eligible responded that:

*“I didn’t buy that idea (CCLAD). I would rather pick them myself because I may wake up with a stomach-ache or headache, come to the facility, and get some Panadol in addition to my ARVs. So, the one picking for you will not know, and even the doctor at the facility will not know” a 47-year-old female.*

##### Privacy concerns

Some PLHIV felt that they could not confide in the group leader private health concerns such as having a sexually transmitted infection (STI). This was due to fears of being stigmatized and the lack of technical capacity of the group leader to manage them. PLHIV explained that they were more comfortably confiding in their healthcare providers, who would keep their secrets. Participants reported that the clients who visited HIV clinics with non-HIV-related complaints were offered treatment, but in the community, one only receives ARVs. This made the model less attractive.

*“What brings most people to the clinic is the care. If I am suffering from something private, there is no way I will tell someone else, but when you come to the facility, they ask if you are suffering from anything, and when you say yes, they check you, treat you, and give you medicine”* – a 54-year-old male.

#### Limited opportunity to negotiate

Not only were respondents concerned that CCLAD would detach them from healthcare providers, but they were also concerned about their ability to negotiate longer refills, which were deemed necessary for mobile participants and those with demanding jobs.

*“Some of us prefer coming to the clinic so that we can explain to the health workers that you will not be available for a long time so that they can add you more,”* – *a* 44 year-old male.

## Discussion

This study explored PLHIV perceptions and factors associated with the uptake of the CCLAD model at Mulago HIV clinic, Kampala, Uganda. We found that only 7.1% of PLHIV used the CCLAD model, which is less than half of the 15% set by the MOH [6]. The factors associated with current enrolment in CCLAD were longer ART history and consistent prior engagement in care with no missed appointments. Our qualitative interviews revealed that PLHIV had concerns about the CCLAD model’s group formation. There were also concerns about being potentially stigmatized, and what they considered to be relatively inferior services at the community level as opposed to those received in the facility-based model. Participants also reported that the CCLAD model could potentially provide avenues for patient networking, and social support, as well as savings on transport costs and time.

The low uptake of the model at 7.1% resonates with three major themes in the qualitative study: anticipated HIV stigma, complex group formation process, and feeling of detachment from health provider interactions. Most of the participants expressed their fear of disclosing their HIV-positive status by being members of a CCLAD group.

The low uptake of CCLAD in this study relates to an earlier Ugandan study that found that 6% of PLHIV used CCLAD [4]. The association between consistent appointment-keeping and CCLAD participation in the quantitative analysis resonates with the social support theme that emerged from the in-depth interviews. Some respondents thought that the CCLAD model facilitated regular clinic attendance through social support and saving transport costs which addresses access barriers, especially in a crisis.

The association between longer duration on ART and consistent appointment keeping with CCLAD participation was also supported by qualitative results in which participants perceived CCLAD as being convenient and saving transport costs. The greater preference for CCLAD by highly ART-experienced PLHIV could be due to the fact that participants on ART for a longer duration are likely to have overcome internalized and anticipated stigma [9]. Participants with a long duration of treatment are also more likely to be stable, have disclosed their HIV status, and have long-term friends. Therefore, such experienced participants can easily form and maintain community-based treatment groups.

The association between long duration on ART and CCLAD uptake also resonates with the findings of in-depth interviews, in which participants felt that CCLAD is better suited for clients who have been on ART for a longer duration as they would easily manage drug side effects individually. Whereas in the quantitative findings, the duration on ART was associated with the possibility of choosing CCLAD because of the intensive adherence support they might have received over time, in the qualitative, the participants had mixed reactions. Some participants expressed optimism for CCLAD, while others reported resentment due to anticipated stigma. This implies that stigma is still a major issue that does not wane with prolonged ART use. Therefore, there is a need for stigma reduction interventions within community ART delivery models.

Overall, PLHIV who did not adopt the CCLAD model had several concerns, such as anticipated stigma due to unintended disclosure of their HIV-positive status to third parties and detachment from facility and health care providers. There were concerns about trusting drug delivery by lay workers, the inability to negotiate for more months of ART refills because of representation, and complexities in the process of group formation.

Participants based on individual concerns and perceptions to choose the CCLAD model. For example, some respondents in the qualitative interviews felt unprepared for the model, raised social stigma concerns, fear of disclosure of status, group dynamics including misunderstanding, and lack of trust in the capacity of lay workers to safely pick, store, and deliver ARVs. PLHIV preferred to conceal their HIV status in the facility-based model. Therefore, these individual perceptions must be addressed for the CCLAD to be appealing.

The study findings suggest the need for practitioners, policymakers, and other stakeholders to prioritize meaningful stakeholder engagement, through dialogues to allay fears and anxiety. The process of enrolment into CCLAD ought to integrate strategies of HIV status disclosure, trust-building, team building, improving group dynamics, peer-led channels of communication, social support, and periodic community-based health care provider visits. The findings challenge the underlying assumption of the DSD model. First and foremost, the assumption of stable PLHIV on treatment can easily accept community-based treatment models. Our results revealed that the majority of stable PLHIV are attached to facility-based models as protective mechanisms against social discrimination. Additionally, some PLHIV perceived the CCLAD model as being complex, incompatible with their needs, inadvertent HIV disclosure and facilitating HIV stigma [5, 10], and offering fewer services relative to the facility-based models. These findings support earlier research in western Uganda [11], which suggested the need for practitioners to mitigate PLHIV from internal stigma and fear of being detached from health workers [5].

### Strengths and limitations of the study

This study highlights the importance of meaningful stakeholders’ engagement in understanding and addressing perceptions that will influence the successful implementation of the CCLAD model. Additionally, this being a mixed methods study, it allowed the exploration of how PLHIV perceptions influence the uptake of the CCLAD model. It is also important to note that the study was conducted in the largest HIV clinic in Uganda, which serves clients from different parts of the country; therefore, the results can be generalized.

### Limitations of the study

This study had some limitations. First, the quantitative analysis was based on secondary data analysis, which is often challenged with missing information. Indeed, 40 participants were excluded from quantitative analysis for missing data. Nonetheless, the sample size was sufficient, and the study was well-powered and ensured- robust results. We determined the uptake of CCLAD using data from participants with complete information. Second, perceptions are subjective, can change over time, and may have changed from when data were collected to when respondents were interviewed. Finally, the cross-sectional design could not allow the determination of causality, although the reported association remains statistically valid.

## Conclusion

The uptake of the CCLAD model at large HIV clinic in Uganda is lower than the national recommended percentage of 15%. Its uptake was associated with those who had been in care for a longer period and who did not miss appointments. The CCLAD model was praised for improving social support, reducing transport costs, and hence improving accessibility to services, linkage, and retention in care. Despite CCLAD being perceived as convenient and promoting social support, several challenges were expressed. These included complexities of group formation, fear of stigma and feelings of detachment from health facilities among others. While CCLAD presents a promising alternative ART delivery model, more attention needs to be paid to the processes of group formation and improved patient monitoring to address the feeling of detachment from the facility. The CCLAD presents an alternative care model for those who are uncomfortable with facility models. In the era of patient-centered care, HIV clinics and programs should address negative perceptions and concerns. Such improvements will be critical in improving HIV patient health outcomes, including access, linkage, and retention in HIV care in Uganda.

### Electronic supplementary material

Below is the link to the electronic supplementary material.

Supplementary Material 1

## Data Availability

The datasets used and/or analyzed during the current study can be available from the corresponding author upon reasonable request.

## Abbreviations

aOR: Adjusted Odds Ratio
ART: Antiretroviral therapy
CCLAD: Community Client-Led ART Delivery
CDDP: Community Drug Distribution Points
DSD: Differentiated Service Delivery
FTDR: Fast-Track Drug Refill
MOH: Ministry of Health
PLHIV: People Living with HIV
TASO: The AIDS Support Organization
UNCST: Uganda National Council for Science and Technology
WHO: World Health Organization
