# Supplementary material for: Perceptions and factors associated with the uptake of the community client-led antiretroviral therapy delivery model (CCLAD) at a large urban clinic in Uganda: a mixed methods study
Source: BMC Health Serv Res. 2023 Oct 26;23:1165. doi: 10.1186/s12913-023-10182-7 (PMC10605330; doi:10.1186/s12913-023-10182-7)
Supplement: Supplementary file 1 — Supplementary Material 1 [file 12913_2023_10182_MOESM1_ESM.docx]

**SUPPLEMENTARY FILE 1: IN-DEPTH INTERVIEW GUIDE FOR PLHIV**

**Study Title: Perceptions and factors associated with the uptake of the community client-led antiretroviral therapy delivery model (CCLAD) at a large urban clinic in Uganda:**

**A mixed methods study.**

**Introduction**

*Thank you for taking the time to speak with me today. My name is [name of interviewer] and I am working on behalf of …………………. We are conducting a qualitative study and would like to ask you a few questions about your thoughts on the community client-led antiretroviral therapy delivery model (CCLAD). Your participation will help us better understand your experience with the CCLAD model, as well as the overall uptake of community models for HIV services in Uganda. We have invited you to participate because you are a client receiving care at this facility, and your views are crucial to this study. During the interview, we encourage you to share your honest opinions and there are no right or wrong answers. The interview will last approximately 45 minutes. Thank you for your time and participation.*

**IDENTIFICATION DATA**

Interview ID: ___________________________

Place of the interview: __________________________________

Language: ____________Date _____________ Start Time: _________

1. Please briefly introduce yourself, including your education, occupation, and interests.
2. What have you heard about the Differentiated service delivery models (DSD) (Probe for Fast track drug refills(FTDR), Facility-based groups(FBG), Community Client ART delivery model(CCLAD)
3. Can you please tell me which ones you're familiar with and how you got to know about them?
4. Can you please explain the process that you followed to receive care using this particular model?
5. How long does it take for you to receive your medication in your current model?
6. What are the advantages and disadvantages of receiving care through CCLAD?
7. We have observed low adoption of the CCLAD care model in this clinic. What factors could be contributing to this?
8. What actions can be taken to increase the utilization of CCLAD?
9. How do you feel about the care you receive in your current differentiated service delivery model (DSDM)?
10. Can you share any benefits or challenges of receiving care in CCLAD?
11. If you were to recommend a friend to this clinic, which Differentiated Service Delivery model would you suggest? (Probe for the model and why?)
12. Has there been any impact on transport before and after joining the model? ( Probe: if he/she had ever missed clinic appointment due to transport.
13. When you consider the DSDM you are currently in, what is your opinion on how your health-related issues are being addressed?

What can be done to increase the number of clients utilizing community DSDM care models?

**Wrap UP:**

We have come to the end of our discussion. Do you have any questions or comments? Is there anything important we haven't discussed?

Thank you for sharing your thoughts with me today. We appreciate your willingness to participate.
